# Supplementary material for: Effects of high intensity interval exercise on cerebrovascular function: A systematic review
Source: PLoS One. 2020 Oct 29;15(10):e0241248. doi: 10.1371/journal.pone.0241248 (PMC7595421; doi:10.1371/journal.pone.0241248)
Supplement: S1 Fig — (DOC) [file pone.0241248.s002.doc]

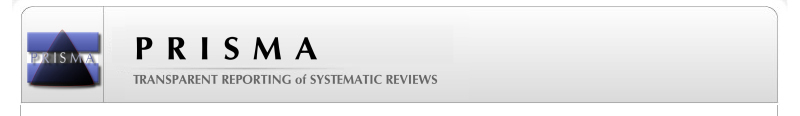
**PRISMA 2009 Flow Diagram**

**Screening**

**Included**

**Eligibility**

**Identification**

Records identified through database searching
(n = 67)

Additional records identified through other sources
(n = 0)

Records after duplicates removed
(n = 20)

Records screened
(n = 20)

Records excluded
(n = 11)

Full-text articles assessed for eligibility
(n = 9)

Full-text articles excluded, with reasons
(n = 2) Not experimental or quasi-experimental study design

Studies included in qualitative synthesis
(n = 7)

Studies included in quantitative synthesis (meta-analysis)
(n = 0)
