# Supplementary material for: Effects of high intensity interval exercise on cerebrovascular function: A systematic review
Source: PLoS One. 2020 Oct 29;15(10):e0241248. doi: 10.1371/journal.pone.0241248 (PMC7595421; doi:10.1371/journal.pone.0241248)
Supplement: S1 Table — (DOCX) [file pone.0241248.s003.docx]

**S1 Table.** Study Exclusion from Systematic Review

|  | **Study** | **Reason for Exclusion** |
| --- | --- | --- |
| **Primary Protocol Not HIIE** | |  |
|  | Dupuy et al. 2019 | Excluded due to a confounding combined intervention of studying the ingestion of different drinks such as a carbohydrate drink, commercial sports drink, glucose, or water. |
|  | Willis et al. 2019 | Excluded due to confounding combined intervention of blood flow restriction. |
|  | Klein et al. 2019 | Excluded due to performing moderate intensity interval exercise rather than high intensity interval exercise. |
|  | Komiyama et al. 2020 | Excluded due to performing high intensity continuous exercise but not high intensity interval exercise. |
|  | Hansen et al, 2020 | Excluded due to confounding combined intervention of studying supplemental carbon dioxide. |
|  | Scott et al. 2019 | Excluded due to confounding combined intervention of studying head down tilt. |
| **No Cerebral Artery Assessment** | |  |
|  | Saoi et al. 2019 | Excluded due to measuring muscle tissue metabolism and not measuring cerebrovascular system. |
|  | Hanssen et al. 2018 | Excluded due to measuring retinal vessels and not directly measuring intracranial cerebrovascular system. |
|  | Gjellesvik et al. 2020 | Excluded due to measuring peak oxygen consumption and not directly measuring cerebrovascular system. |
|  | Kirkham et al. 2017 | Excluded due to measuring cardiac function and not directly measuring cerebrovascular system. |
| **Animal Study** |  |  |
|  | Peyravi et al.2020 | Excluded secondary to animal (no human) study. |
| **Non-Experimental Study Design** | |  |
|  | Labrecque et al. 2019 | Excluded observational studies |
|  | Tsukamoto et al. 2019 | Excluded observational studies |
